# Mitochondria transfer restores fibroblasts-like synoviocytes (FLS) plasticity in LPS-induced, in vitro synovitis model

**DOI:** 10.1186/s12964-022-00923-2

**Published:** 2022-09-07

**Authors:** K. Kornicka-Garbowska, S. Groborz, B. Lynda, L. Galuppo, K. Marycz

**Affiliations:** 1 Department of Experimental Biology, Wroclaw University of Environmental and Life Sciences, Norwida 27B Street, A7 Building, 50-375 Wroclaw, Poland; 2 International Institute of Translational Medicine, Malin, Jesionowa 11, 55-114 Wisznia Mała, Poland; 3 Department of Surgical and Radiological Sciences, School of Veterinary Medicine, University of California Davis, Davis, CA USA

**Keywords:** Synovitis, Synoviocytes, Mitochondria, Mitochondria transfer

## Abstract

**Background:**

Synovitis (SI) is one of the most common and serious orthopedic diseases in horses of different age, breed and sex, which contributes to the development of osteoarthritis. The burden of SI includes economic loss and represents a real challenge for current veterinary health care. At the molecular level, fibroblasts-like synoviocytes (FLS) are recognized as major cell populations involved in SI pathogenesis. In the course of SI, FLSs are losing their protective and pro-regenerative cytological features, become highly proliferative and initiate various stress signaling pathways.

**Methods:**

Fibroblast-like synoviocytes were treated with LPS in order to generate SI in vitro model. Mitochondria were isolated from peripheral blood derived mononuclear cells and co-cultured with FLS. After 24 h of culture, cells were subjected to RT-qPCR, western blot, cytometric and confocal microscopy analysis.

**Results:**

Mitochondrial transfer (MT) was observed in vitro studies using confocal microscopy. Further studies revealed, that MT to LPS-treated FLS reduced cell proliferation, modulated apoptosis and decreased inflammatory response. Overall, MT Resulted in the considerable recovery of recipient cells cytophysiological properties.

**Conclusions:**

Presented data provides evidence that mitochondria transfersignificantly modulate FLS proliferative and metabolic activity through improved mitochondrial biogenesis and dynamics in activated FLS. Obtained results for the first time demonstrate that horizontal MT might be considered as a therapeutic tool for synovitis treatment; however, further clinical studies are strongly required.

**Video abstract**

**Supplementary Information:**

The online version contains supplementary material available at 10.1186/s12964-022-00923-2.

## Background

Synovitis (SI) is one of the most common orthopedic diseases in horses of different ages, breeds and sex, and is recognized as a one of the critical factors that contributes to the development of osteoarthritis (OA). There are several initiating factors of SI including trauma, hemarthrosis, poor joint conformation, and joint infections that cause different grade of lameness, which eliminates horses from their further activity and requires costly pharmaceutical intervention [1]. SI due to joint effusion and lameness is recognized as a major cause of decreased performance in horses that is associated with serious financial loss for the equestrian industry. The current available treatment methods include lavage of the affected synovial cavity to clear pathogens, toxins, and inflammatory mediators combined with systemic application of non-steroidal anti-inflammatory drugs, as well as intraarticular administration of steroids and lubricants [2]. Although pain and inflammation attenuation can be achieved, these treatments remain ineffective in stopping disease progression and therefore there is an urgent need to develop more efficient therapeutic solution for SI treatment.

The histological architecture of synovium in the course of SI is characterized by synovial lining hyperplasia, hyper vascularization and proinflammatory cells hyper activity. At the molecular level, fibroblasts-like synoviocytes (FLS, CD34−THY1−, CD34−THY1+, CD34) and macrophages-like synoviocytes (MLS, CX3CR1-AQP1) are recognized as major cells populations involved in synovitis and in humans osteoarthritis (OA) pathogenesis [3]. Under physiological condition, FLS are responsible for the secretion of hyaluronan and lubricin to synovial fluid and thus protects cartilage from injury, while MLS maintain immunometabolism homeostasis. In the course of SI, FLSs are losing their protective and pro-regenerative cytological features, and become highly proliferative and metabolically active cells initiating various stress signaling pathways. Activated FSL have been shown to produce a broad range of proinflammatory cytokines including IL-1β, IL-6, IL-9, IL-15 or TNF-α while losing their immunomodulatory effect [4]. Recent data have demonstrated that activated FLS additionally release a wide plethora of caspases and metalloproteinases, that initiate unreversible joint degenerative changes. Moreover, activated FLS secrete specific reactive metabolites related to gluconeogenesis, amino acids metabolism, and lipolysis, that maintain their proliferative and highly aggressive phenotype [5]. Furthermore, activated FLS secrete fibroblast activation protein-α (FAPα) that enhances inflammation and bone erosion, which is recognized as a major contributor of OA development [6]. In the course of SI, FLS switch their quiescent phenotype towards an aggressive and highly proliferative one and subsequently activate macrophage-like synoviocytes (MLS), that secrete abundant amounts of proinflammatory mediators, leading to increased hypoxia, oxidative stress, impaired angiogenesis and synovial fluid nutrient depravation [7]. Once MLS are activated, they promote matrix degrading through the secretion of vast amount of TNF-α and IL-1β that shift the anabolic homeostasis of synovial tissue in favor of increased catabolism [8]. The highly inflammatory microenvironment of synovium, excessive oxidative stress combined with nutrient depravation trigger to mitochondrial metabolism failure, initiating FLS apoptosis and joint degeneration. Both inflammation and abundant oxidative stress are directly associated with FLS mitochondrial bioenergetics disruption, defective mitophagy and diminished superoxide dismutase (Mn-SOD) activity. Mitochondrial dysfunction of activated FSL is mainly associated with the loss of mitochondrial membrane potential, resulting in collapsed energy production and increased mitochondrial outer membrane permeabilization (MOMP) that lead to the release of apoptogenic molecules such as cytochrome c, Smac/Diablo, HtrA2 (Omi), apoptosis-inducing factor (AIF) and procaspases from the mitochondrial intermembrane space into the cytoplasm [9, 10]. The molecular deterioration of mitochondrial biogenesis and dynamics together with accumulated damaged mtDNA could be directly involved in excessive generation of mROS and NRS initiating the transition of FLS to a defective phenotype.

One of the critical mechanisms that maintain tissues immunometabolic homeostasis lies in the natural horizontal mitochondrial transfer (MT) between progenitor and neighboring cells including macrophages. The discovery of MT from mesenchymal stromal cells (MSCs) to recipient cells aroused great interest in the field of regenerative medicine [11]. This unique mechanism allows to replace dysfunctional mitochondria and restore them with their healthy counterparts to prevent apoptosis, inflammation and oxidative stress that in consequence might serve as a new therapeutic tool. Recently, MSCs have been reported to transfer their mitochondria into macrophages, allowing to restrain their pro-inflammatory functions. It was demonstrated that MSCs maintain tissue microenvironment and immunometabolic balance by improving donor macrophages mitochondrial metabolism and dynamics [12]. This in turn promotes M2 macrophages polarization and might be recognized as a critical immunomodulatory mechanism that protects tissue from inflammation and resulting injury. Recent study demonstrated, that in response to reactive oxygen species (ROS) overproduction and damage-associated molecular patterns (DAMPs) including defective and mutated mtDNA accumulation, MSCs cells tend to donate healthy mitochondria to injured cells [13]. Transferred mitochondria through mitochondrial fusion subsequently enhance donor cells ATP production, improve oxidative phosphorylation, and thus protect against cells apoptosis [14].

In this study we were interested in elucidating whether mitochondrial transfer restores FLS plasticity in an in-vitro experimental model of LPS-induced synovitis. We have found that MT significantly modulate FLS proliferative and metabolic activity through improved mitochondrial biogenesis and dynamics in activated FLS. Obtained results for the first time demonstrate that horizontal MT might be considered as a therapeutic tool for synovitis treatment; however, further clinical studies are strongly required.

## Materials and methods

### Fibroblast like synoviocytes (FLS) isolation

Samples of synovial tissue were collected from horses in the slaughter house (n = 6). The synovium was separated with scissors into small pieces and transferred into tube containing DMEM and 1% type IV collagenase. Subsequently, the specimens were incubated at a constant temperature of 37 °C for 60 min. Following completion of the incubation, samples were centrifuged for 5 min at 300× *g* and resuspended with DMEM supplemented with 10% FBS and 1% penicillin–streptomycin. The cells were seeded cells into a 75-cm2 flask and placed in a humidified tissue culture incubator (37 °C, 5% CO2).

### Mitochondria isolation

Mitochondria were isolated from horses blood with Mitochondria Isolation Kit for Cultured Cells (Thermo Scientific™) in according to instructions provided by manufacturer. Blood were collected into tube with sodium citrate. PBMCs were dissolved with Reagent A. Cells were incubated 2 min on ice. Then the Reagent B were added and incubated on ice for 5 min, vortexing every one minute. Solution were centrifuged. Obtained pellet were dissolved in Reagent C and centrifuged. Obtained mitochondria were washed with PBS, then the concentration were measure with BCA Assay Kit (Thermo Scientific™) and used for further experiments.

### Experimental model

Synoviocytes were seeded onto a 24-well plates at a density of 25 × 10^4^ and were cultured in Dulbecco's Modified Eagle's Medium (DMEM) containing 1 g/L glucose, supplemented with 10% fetal bovine serum (FBS) and 1% penicillin–streptomycin antibiotic solution. Cells were cultured in an incubator with 5% CO_2_ and 95% humidity at 37 °C.

SI in vitro inflammation model in FLS was induced by LPS. Cells were treated with 1 µg/mL LPS for 5.5 h. The medium was then replaced with the medium containing various doses of mitochondria (MT1 = 8 µg per 100,000 cells, MT2 = 200 µg per 100,000 cells). After 24 h cells were collected and subjected for further analysis. Cells cultured in DMEM without LPS served as a negative control group (CTRL) while treated with LPS only (LPS) as a positive control.

### Proliferation rate assay

Cells viability were evaluated with MTS Assay (Abcam) in accordance with the instructions provided by the manufacturer. The experiments were conducted in three different conditions and times (T0—24 h after seeding, before treatment with LPS; T1—24 h after treatment with 1 μg/mL LPS; before treatment with mitochondria; T2—24 h after treatment with mitochondria) then the 20 µl of MTS Reagent was added per well, cells were incubated for 2 h at 37 °C, and then absorbance was measured at 490 nm.

In order to test the ability of cells to form colonies, a clonogenic assay was performed. For this purpose, cells were seeded onto a 6-well plate at a density of 1 × 10^2^. The cells were treated with the LPS or/and mitochondria (MT1/MT2). After 7 days of incubation, cells were fixed with cold 4% PFA for 40 min in the dark at room temperature and then colonies were stained with pararosaniline for 5 min. Cells were washed with PBS 3 times. A series of photos were taken. Colony forming unit fibroblastic assay (CFU-Fs) was calculated using the formula described by Kornicka et Al. [15].

### Visualization of cells’ mitochondria and cytoskeleton

Mitochondria were stained with MitoRed dye (1:1000 in cell medium) on viable cells for 30 min in the dark at 37 °C. The medium containing the MitoRed dye was removed and the cells were washed three times with PBS. Cells were fixed with 4% PFA for 40 min in the dark at room temperature. Cells were permeabilized with 0.1% Triton X-100 solution for 20 min. The cytoskeleton was stained using atto-488-labeled Phalloidin (1:800 in PBS) for 45 min in the dark at room temperature. Cell nuclei were stained with DAPI (Faramount Aq Mounting Medium, Dako). A series of photos was taken with confocal microscope (Observer Z1 Confocal Spinning Disc V.2 Zeiss) and analyzed with ImageJ Software. In order to visualize the incorporation of exogenous mitochondria into recipient cells, mitochondria of PBMCs prior isolation were stained with MitoRed dye for 30 min in the dark at 37 °C.

### Visualization of Ki-67 accumulation

Untreated and treated cells with LPS or/and mitochondria (MT1/MT2) were fixed with 4% PFA as described above. Cells were blocked with 5% BSA and 22 mg of glycine for 1 h at 4 °C and incubated overnight with the Ki-67 antibody (1:100, Abcam). Cells were washed with PBS 3 times following incubation with atto-594 secondary antibody (1:1000, Sigma). The cell nucleus was stained with DAPI. Cells were observed with a confocal microscope and the obtained data was analysed using Image J Software.

### Immunostaining with Caspase 3

To visualize apoptotic processes in treated and control cells, immunostaining with Caspase 3 (Casp-3) antibody was performed. The cells were fixed in 4% PFA for 40 min in the dark as described above. Cells were washed with PBS containing 2% FBS and then permeabilized with 0.05% Triton x-100 in PBS for 15 min in room temperature. Samples were blocked in 5% normal goat serum in PBS for 1 h. Cells were incubated with Casp-3 primary antibody overnight in 4 °C (Thermo Scientific, 1: 100). The cells were then incubated with the goat anti-mouse IgG conjugated to Atto-629 secondary antibody (Thermo Scientific, 1: 500) for 1 h at 37 °C. Cell nuclei were stained with DAP.

### Gene expression analysis

Total RNA was isolated with the EXTRAzol reagent (Blirt, Gdańsk) following manufacturer’s instructions. The concentration, quality as well as purity were measured using a nanospectrophotometer (Epoch, BioTek). Transcription of RNA into cDNA was performed using the Takara PrimeScriptTM RT Reagent Kit with gDNA Eraser (Perfect Real Time). Quantitative Real-Time PCR (RT-qPCR) was performed using the SensiFast SYBR and Fluorescein Kit (Bioline, London, UK) according to the instructions provided by manufacturer. The Real-Time PCR was performed with a CFX ConnectTM Real-Time PCR Detection System (Bio-Rad). The Real-Time PCR program were conducted as follow: 95 °C for 2 min followed by 41 cycles at 95 °C for 15 s, annealing for 30 s and elongation at 72 °C for 15 s. The qPCR results were replicated in 3 independent experiments, and then the statistics were determined. Relative gene expression was normalized by the reference gene the glyceraldehyde 3-phosphate dehydrogenase (GAPDH) using the 2−ΔΔCT method. Primers used are shown in Table 1.

**Table 1:** Sequences of primers used in qPCR

Gene	Primer Sequence (5′->3′)
BAX	F: GGCACCTCTTCCCTCCTTTCT
	R: CGATGCGCTTGAGACACTCG
BCL2	F: TTCTTTGAGTTCGGTGGGGT
	R: GGGCCGTACAGTTCCACAA
p21	F: GAAGAGAAACCCCCAGCTCC
	R: TGACTGCATCAAACCCCACA
p53	F: TACTCCCCTGCCCTCAACAA
	R: AGGAATCAGGGCCTTGAGGA
IL-1β	F: TATGTGTGTGATGCAGCTGTG
	R: ACTCAAATTCCACGTTGCCC
IL-6	F: CGTCACTCCAGTTGCCTTCT
	R: GCCAGTACCTCCTTGCTGTT
TNF α	F: TCCTACCCGTCCAAGGTCAA
	R: CTCATACCAGGGCTTGGCTT
IL-10	F: CTAGGGAACGAAGCATCCAGG
	R: TCAGGAGAGAGGTACCACAGG
IL-18	F: CGCACCCCAGACCGTATTTA
	R: CGCTAGACCTCTAGTGAGGC
CFLAR	F: CCACTTAGGAAACAGCTGCC
	R: AGGCCTTGAGGCTACTGACT
CRSL1	F: TGGGCCAGTCAAAAATCAGC
	R: TTCGCGGTGTTGGAAGAGTT
MALT1	F: ATGATGTGCGACGCCTATGT
	R: AGCTGCCAGTTTCTTCAGGT
CARD9	F: CAGCAACTCCTGTGGCTCAT
	R: GCACTCGTCCTCGTTCTCAT
NLRP3	F: CGACACTCTGACATGGACCG
	R: GGCTGAGAAGAGGCTCTGAC
MAP2K1	F: ACTGGGAAAGCTTGGGATGC
	R: CAGTTCCCCAACCTTCTGCT
MAP2K2	F: CGCAGGACTTCCAGGAGTTT
	R: CATCTTCAGATCCGCTCGCT
MAPK8	F: GTTGGGTGCATCATGGGAGA
	R: CAGGACATGGTGTTCCAAGC
MAPK14	F: GAACGTTGTTTCCTGGCACA
	R: TCTTGCAGACTCTGAGGAGATTT
NFKB	F: CTTCCTTCGAGCCAGTGACG
	R: CCAGGAGACTTGCTGTCGTG
IKK	F: GGCGGTTGACATTAGCACAG
	R: CTGAAGCCGAACCACAGTCT
NFKBIA	F: CACTTCACCTTCGTGAGGCT
	R: TGTCACAGGACACAACTGGG
TLR1	F: GTGAGTGGTGCCATTATGAG
	R: CGCGTTTAGATTCAGGTC
TLR2	F: TCGCCGGGACTCTCTTTCTC
	R: GGCCTTGAGGTTCACACACT
TLR3	F: GACTGATGCTCCGAAGGGTG
	R: GGTTTGCGTGTTTCCAGAGC
PGC1A	F: GGCCTTCTAAACGTGGGACA
	R: CCGGAGGTCTGCCATTTTCT
FIS1	F: GGTGCGAAGCAAGTACAACG
	R: GTTGCCCACAGCCAGATAGA
MFN1	F: AAGTGGCATTTTTCGGCAGG
	R: TCCATATGAAGGGCATGGGC
MFN2	F: AGGTGAAGTCAGAATTGGTGGA
	R: CTTCACAGGGGTGGCATCAT
Parkin	F: TCCCAGTGGAGGTCGATTCT
	R: CCCTCCAGGTGTGTTCGTTT
PINK1	F: GCACAATGAGCCAGGAGCTA
	R: GGGGTATTCACGCGAAGGTA
GAPDH	F: GATGCCCCAATGTTTGTGA
	R: AAGCAGGGATGATGTTCTGG

*BAX* BCL-2-associated X protein, *BCL-2* B-cell lymphoma 2, *p21* cyclin-dependent kinase inhibitor 1A, *p53* tumor suppressor p53, *IL-1β* Interleukin1β, *IL-6* Interleukin 6, *TNFα* tumor necrosis factor, *IL-10* interleukin 10, *IL-18* interleukin 18, *CFLAR* CASP8 and FADD-like apoptosis regulator, *CRSL1* cardiolipin synthase 1, *MALT1* mucosa-associated lymphoid tissue lymphoma translocation protein 1, *CARD9* Caspase recruitment domain-containing protein 9, *NLRP3* NLR family pyrin domain containing 3, *MAP2K1* dual specificity mitogen-activated protein kinase kinase 1, *MAP2K2* mitogen-activated protein kinase kinase 2, *MAPK8* mitogen-activated protein kinase 8, *MAPK14*: mitogen-activated protein kinase 14, *NFKB* nuclear factor kappa B, *IKK* IkappaB kinase, *NFKBIA* nuclear factor kappa-B inhibitor, *TLR1* toll-like receptor 1, *TLR2* toll-like receptor 2, *TLR3* toll-like receptor 3, *PGC1A* peroxisome proliferator-activated receptor-gamma coactivator, *FIS1* mitochondrial fission 1 protein, *MFN1* mitofusin-1, *MFN2* mitofusin-2, *MFF* mitochondrial fission factor, *PARKIN* E3 ubiquitin ligase parkin, *PINK1* PTEN induced kinase 1, *GAPDH* glyceraldehyde 3-phosphate dehydrogenase

### Western blot analysis

Cells were detached from culture flasks and incubated on ice with RIPA buffer containing 1: 1000 Protease and Phosphatase Inhibitor (Sigma Aldrich, Poznan, Poland). Protein concentration was measured using the Pierce™ BCA Protein Assay Kit (Thermo Scientific™). A final concentretion of 25 µg of proteins was mixed with 4 × Laemelli buffer containing β-mercaptoeatnol. The samples were incubated for 5 min at 95 °C. Prepared proteins were used in the Western Blot.

After SDS-PAGE electrophoresis, proteins were transfer onto polyvinylidene difluoride (PVDF) membranes (Bio-Rad, USA) and then blocked in a 5% non-fat milk solution in TBST for 1 h at room temperature. The different membranes were incubated with primary antibodies overnight (Table 2). Primary antibodies—listed in Table 2—were removed and the membranes washed with 1 × TBST 5 times. Membranes were incubated for 1 h at room temperature with HRP-conjugated secondary antibodies (dilution 1: 1000 in TBST). The chemiluminescent signals were monitored with the ChemiDoc MP Imaging System (Bio-Rad, USA).

**Table 2:** List of antibodies used in study

Antibodies	Concentrations	CAT numbers	Company
β actin	1:1000	orb10033	Biorbyt
IL-1β	1:1000	ab9722	Abcam
IL-6	1:1000	ab6672	Abcam
MFN1	1:1000	orb11040	Biorbyt

*IL-1β* interleukin 1β, *IL-6* interleukin-6, *MFN1* mitofusion 1

### Microcapillary cytometry

Cells were analyzed with commercially available kits using Muse™ Cell Analyzer (Merck, Germany). Cell cycle analysis was analyzed using the Muse™ Cell Cycle kit (Millipore) in accordance with the instructions provided by the manufacturer. As recommended by the manufacturer, cells were suspended in ice cold 70% ethanol then incubated in the dark at − 20 °C overnight. Cells were centrifuged at 300× *g* for 5 min and washed once with 1× PBS. The proper volume of Muse Cell Cycle Reagent was added to the cells, and then cells were incubated for 30 min in the dark at room temperature and subjected to cell cycle analysis. Intracellular oxidative stress factors have been tested using the Muse^®^ Oxidative Stress kit (Luminex) according to the instructions provided by the manufacturer. Prior analysis, cells were incubated at 37 °C for 30 min in Muse^®^ Oxidative Stress working solution. To evaluate cell apoptosis and cell viability, the Muse^®^ Annexin V & Dead Cell Kit (Millipore) was used according to the instructions provided by manufacturer. Prior analysis, cells were incubated with Muse^®^ Annexin V & Dead Cell reagent in the dark for 20 min at room temperature. To evaluate changes in mitochondrial potential and cellular plasma membrane permeabilization, The Muse^®^ MitoPotential Assay Kit (Luminex) was used in accordance with the instructions provided by the manufacturer. The proper amount of cells were diluted in 1× Assay Buffer. Cells were incubated with MitoPotential working solution for 20 min in 37 °C. Then Muse 7-AAD were added to the solution and incubated for 5 min at room temperature. Following incubation, cells were analysed with Muse ™ Cell Analyzer.

### Statistical analysis

The obtained results were analyzed by one way variance analysis (ANOVA) using GraphPad Software 8 (San Diego, USA) and post-hoc Tukey's test. Statistically significant results were marked with an asterisk or number sign, respectively, for: *p* < 0.05 (*,#), *p* < 0.01 (**,##) and *p* < 0.001 (***,###). Results are presented as statistical mean SD from at least three independent experiments. Statistical significance indicated as asterisk (*) when comparing the result to LPS and as number sign (#) when comparing to CTRL.

## Results

### Visualization of incorporated mitochondria

**Fig. 1:**
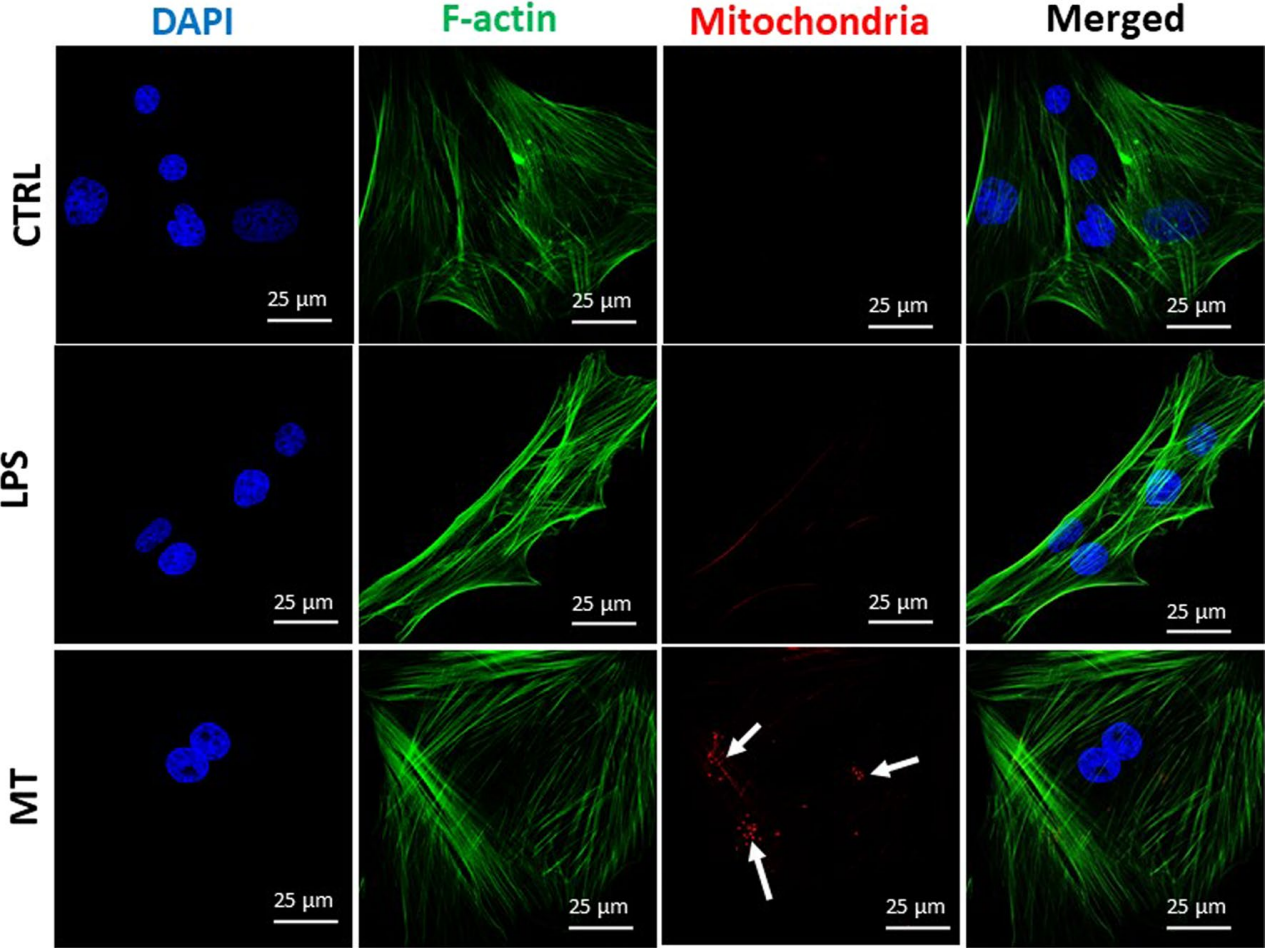
Visualisation of mitochondria in control and experimental group via confocal microscopy. The white arrows show incorporated mitochondria in MT group

In order to visualize mitochondria incorporation, exogenous mitochondria were stained with MitoRed dye, isolated and added to FLS culture medium (Fig. 1). Obtained data clearly showed the present of single mitochondria in MT group (red points indicated with white arrows).

### Evaluation of morphology and viability

**Fig. 2:**
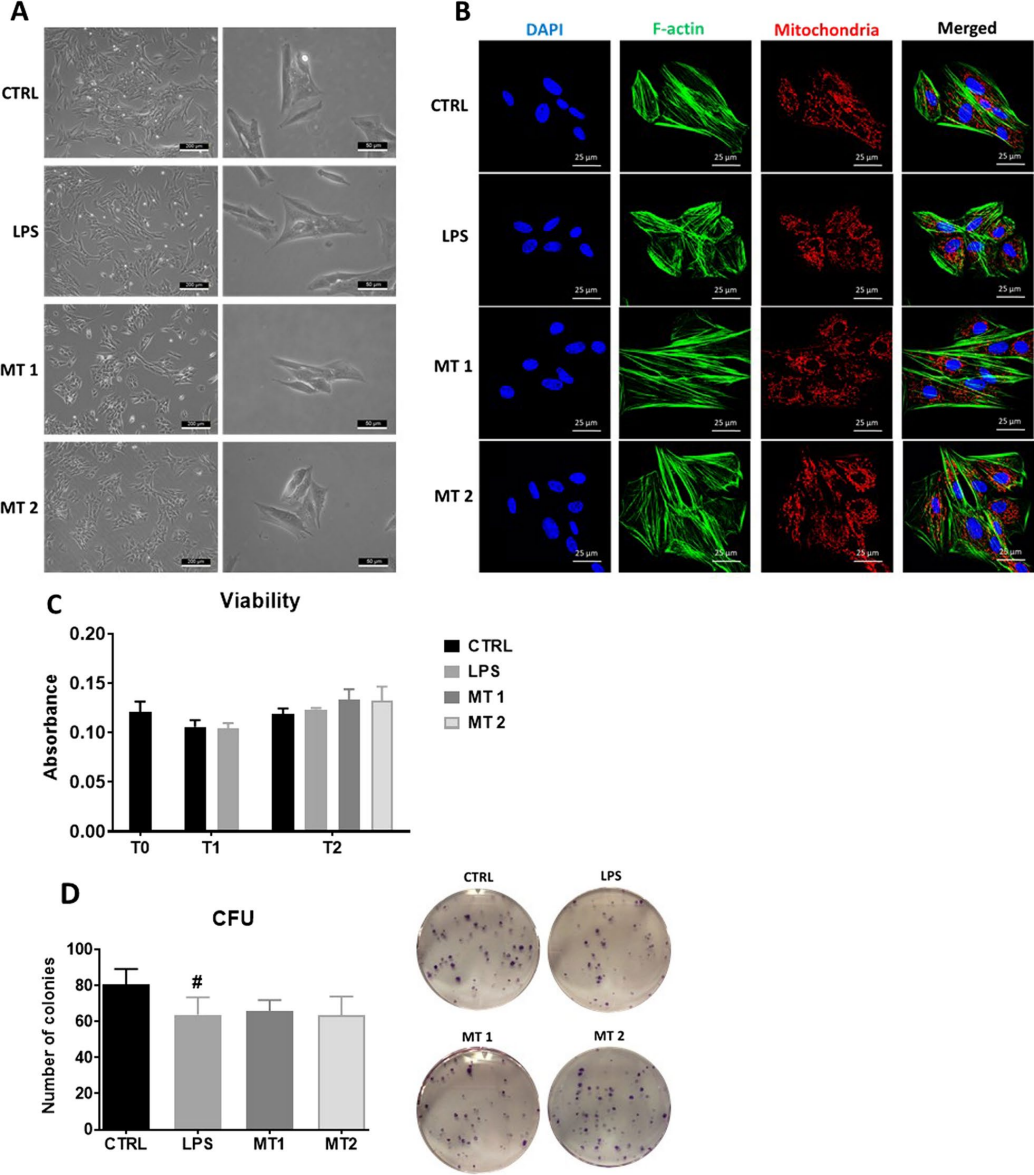

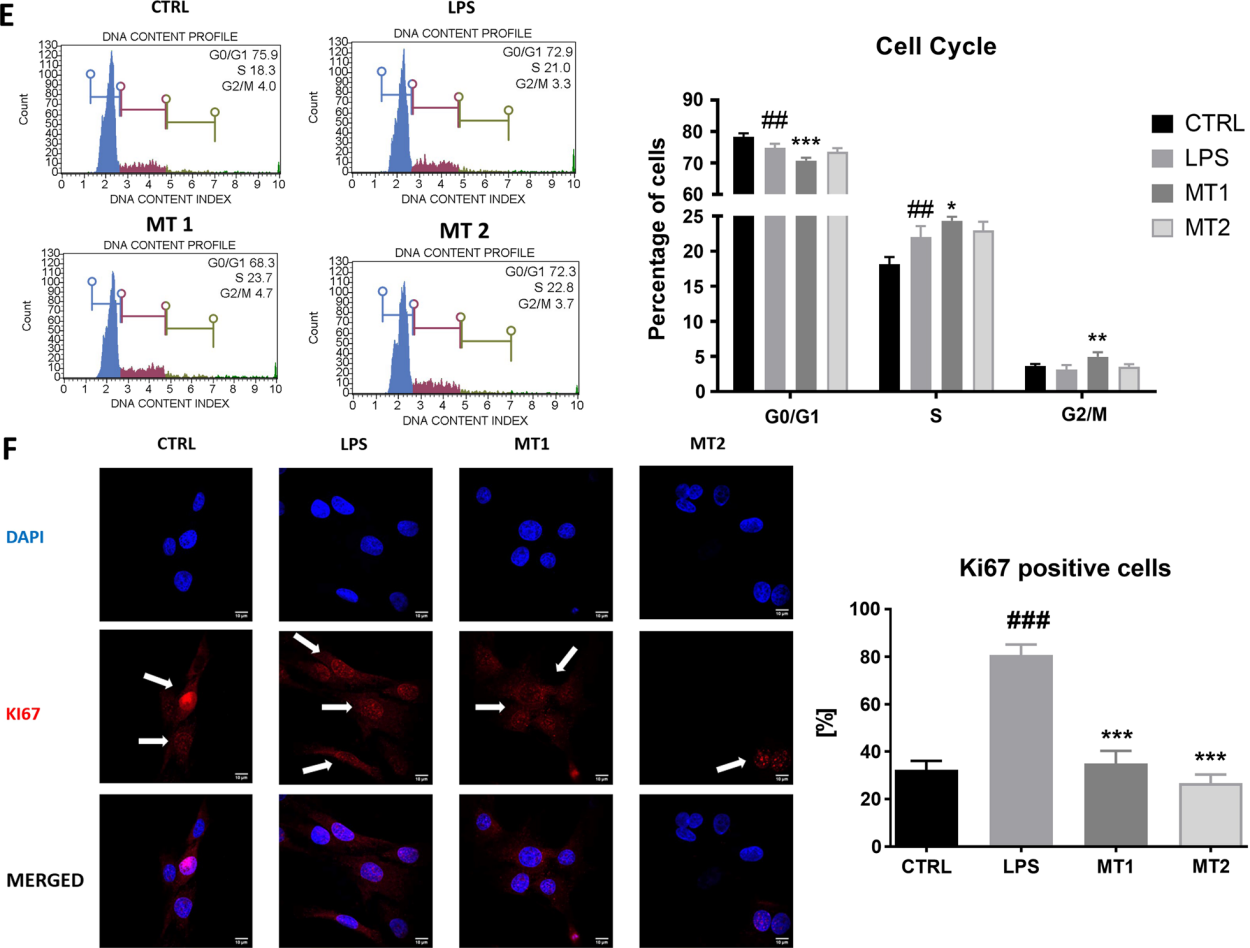
Evaluation of cell morphology with brightfield (**A**) and confocal microscopy (**B**). Cell viability analysis (**C**) and clonogenic assay (**D**) was performed. The Muse^®^ Cell Cycle analysis allowed to analyse the percentage of cells in different cell cycle phases (**E**). Immunostaining with Ki-67 antibody revealed its overexpression in LPS group and reversion of that phenomenon after mitochondrial transfer (**F**). Results expressed as mean ± SD. Statistical significance indicated as asterisk (*) when comparing the result to LPS and as number sign (#) when comparing to CTRL. *,#*p* < 0.05, **,##*p* < 0.01, ***,###*p* < 0.001

Cells morphology was visualised using brightfield (Fig. 2A) and confocal microscopy (Fig. 2B). Obtained data showed no significant differences in cell morphology. However we found that cells in LPS group characterized by "flattened" shape in comparison to MT1 and MT2 group. Moreover, immunostaining analysis of cell morphology revealed changes in F-actin in cells treated with LPS in compared to untreated group. Interestingly, mitochondria application restored regular cytoskeleton network, which became more elongated and well-developed. The same phenomena was observed in mitochondria network in treated and untreated cells. Additionally, we performed cell viability analysis (Fig. 2C). We found, that LPS application decreased number of live cells, however mitochondrial treatment increased viability- although obtained result was not statistically significant. To support cell viability data, we additionally conducted clonogenic assay (Fig. 2D). We noted that in LPS group the number of colonies were lower in comparison to control group. Similar phenomena was observed for MT1 as well as MT2 group.

To support cell viability tests, the Muse^®^ Cell Cycle analysis was performed (Fig. 2E). We found the decreased number of cells in G0/G1 phase in LPS and MT1 groups. Interestingly in MT2 treated group, there was no difference in amount of cells in stationary phase in comparison to LPS group. LPS treatment and MT1 increased the number of cells in S phase. The greatest number of cells in G2/M phase was observed in MT1 group. Immunofluorescence staining for Ki-67 (Fig. 2F) revealed it increased synthesis in LPS treated cells, however mitochondrial application significantly decreased the number of KI-67 positive cells in both experimental groups.

### Evaluation of apoptosis and senescence

**Fig. 3:**
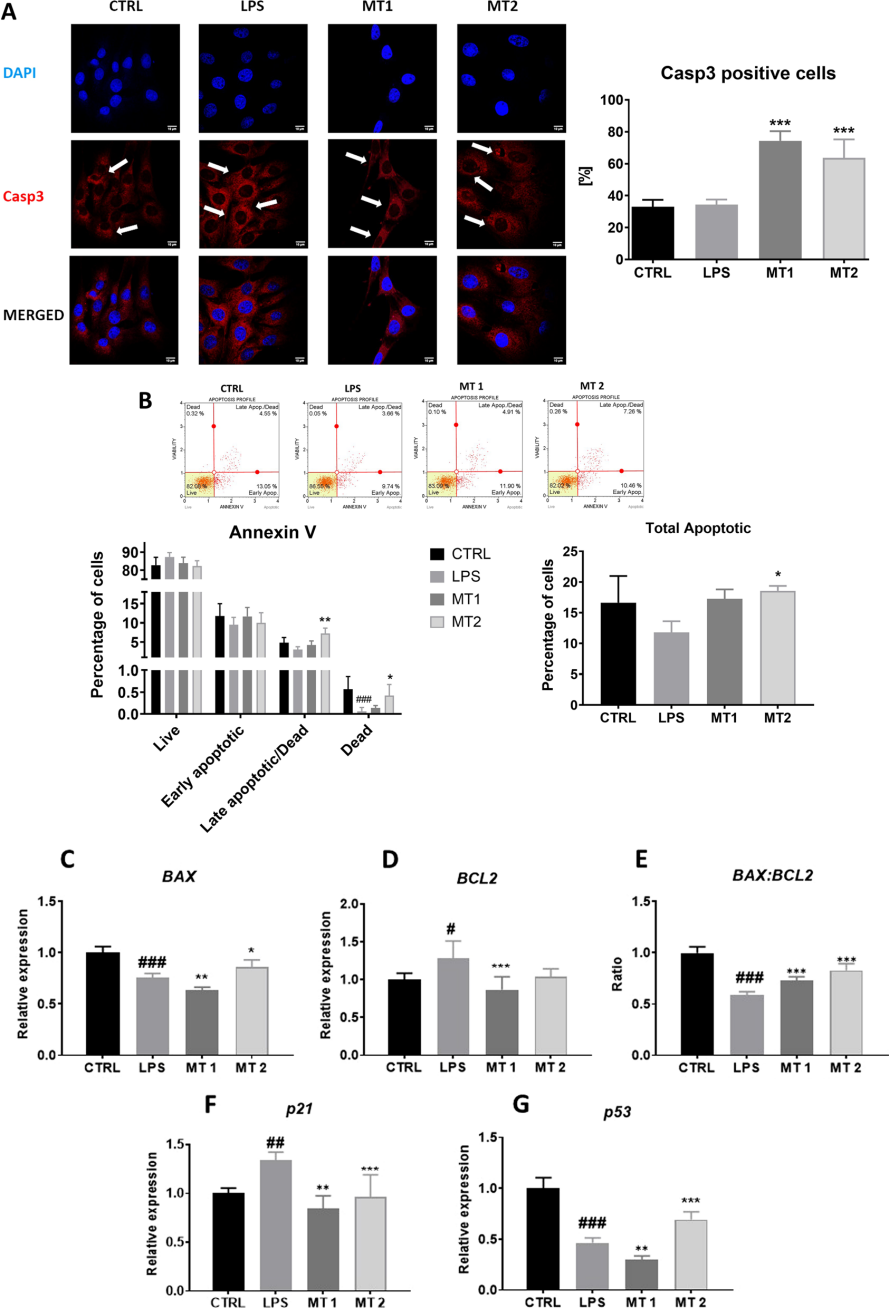
Apoptosis and senescence. The results of Casp-3 immunostaining (**A**). The Muse Muse^®^ Annexin V and Dead Cell analysis revealed the percentage of live, dead and apoptotic cells (**B**). The relative expression Bax (**C**), BCL2 (**D**), the BAX:BCL2 ratio (**E**), p21 (**F**) and p53 (**G**) was established with RT-qPCR. Results expressed as mean ± SD. Statistical significance indicated as asterisk (*) when comparing the result to LPS and as number sign (#) when comparing to CTRL. *,#*p* < 0.05, **,##*p* < 0.01, ***,###*p* < 0.001

To establish whether application of exogenous mitochondria modulate cell apoptosis and senescence, immunostaining for Casp-3 was performed (Fig. 3A). We observed increased number of Casp-3 positive cells in cells after mitotransfer. To support immunostaining data, Muse^®^ Annexin V & Dead Cell analysis was performed (Fig. 3B). Obtained data showed that there was no significant difference in the number of live cells between control as well as experimental groups. The number of total apoptotic cells was the highest in MT2 group. RT-qPCR analysis revealed that LPS application reduced BAX (Fig. 3C) while increased BCL2 expression (Fig. 3D). BAX:BCL2 ratio was increased in both MT1 and MT2 groups (Fig. 3E). Mitochondrial transfer reduced the expression of p21 (Fig. 3F) and p53 (Fig. 3G) but only at MT1 concentration.

### Inflammatory phenotype

**Fig. 4:**
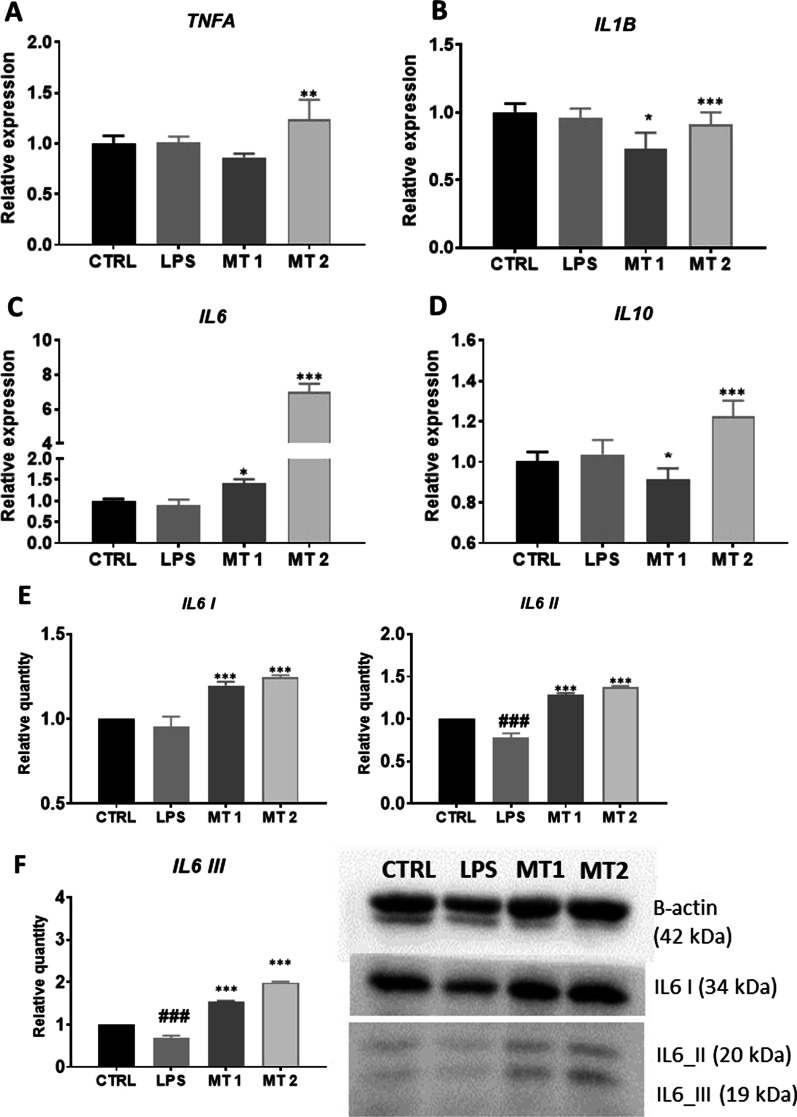
Expression of TNFa (**A**), IL1B (**B**), IL6 (**C**), and e IL10 (**D**) was performed with RT-qPCR. The amount of IL1B (**E**) and IL6 (**F**) isoforms was established with western blot. Results expressed as mean ± SD. Statistical significance indicated as asterisk (*) when comparing the result to LPS and as number sign (#) when comparing to CTRL. *,#*p* < 0.05, **,##*p* < 0.01, ***,###*p* < 0.001

In order to establish inflammatory phenotype, RT-qPCR and Western Blot analysis were conducted. We observed increased expression of TNFA (Fig. 4A) and IL1B (Fig. 4B) in MT2 in comparison to LPS group. Mitochondrial transfer significantly increased the expression of IL-6 (Fig. 4C) in recipient FLS. We also noted enhanced expression of IL10 (Fig. 4D) in MT2 group while comparing to LPS. To support RT-qPCR results, the western blot analysis was performed. Obtained data revealed that mitotransfer reduced the amount of IL1B II cleaved/mature form at both concentrations (Fig. 4E) in comparison to cells treated with LPS only. Interestingly, cells treated with mitochondria displayed enhanced levels of all IL6 isoforms while comparing to LPS group (Fig. 4F).

### Mitogen-activated protein kinases (MAPKs)

**Fig. 5:**
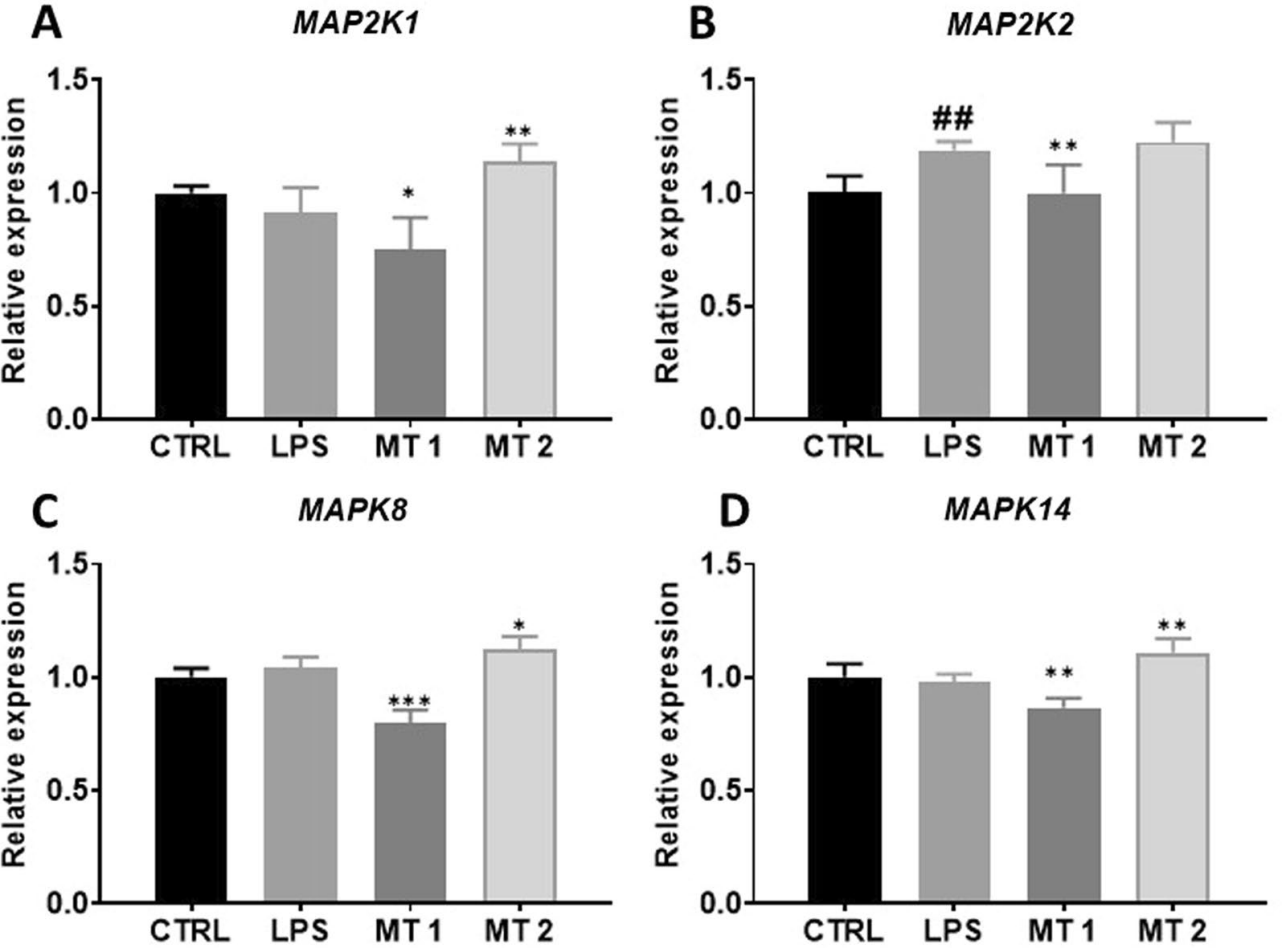
Expression of Mitogen-activated protein kinases (MAPKs). Transcript levels of MAP2K1 (**A**), MAP2K2 (**B**), MAPK8 (**C**) and MAKP14 (**D**) were investigated with RT-qPCR. Results expressed as mean ± SD. Statistical significance indicated as asterisk (*) when comparing the result to LPS and as number sign (#) when comparing to CTRL. *,#*p* < 0.05, **,##*p* < 0.01, ***,###*p* < 0.001

To determine relative expression of MAPK gene complex, RT-qPCR was performed. MAP2K1 expression was upregulated in MT2 group (Fig. 5A) while MAP2K2 reduced in MT1 group (Fig. 5B). Decreased expression of MAPK8 (Fig. 5C) was observed in MT1 while increased in MT2 group in comparison to LPS treated cells. Similar phenomenon was observed for the expression of MAPK14 (Fig. 5D).

### Inflammasome activation

**Fig. 6:**
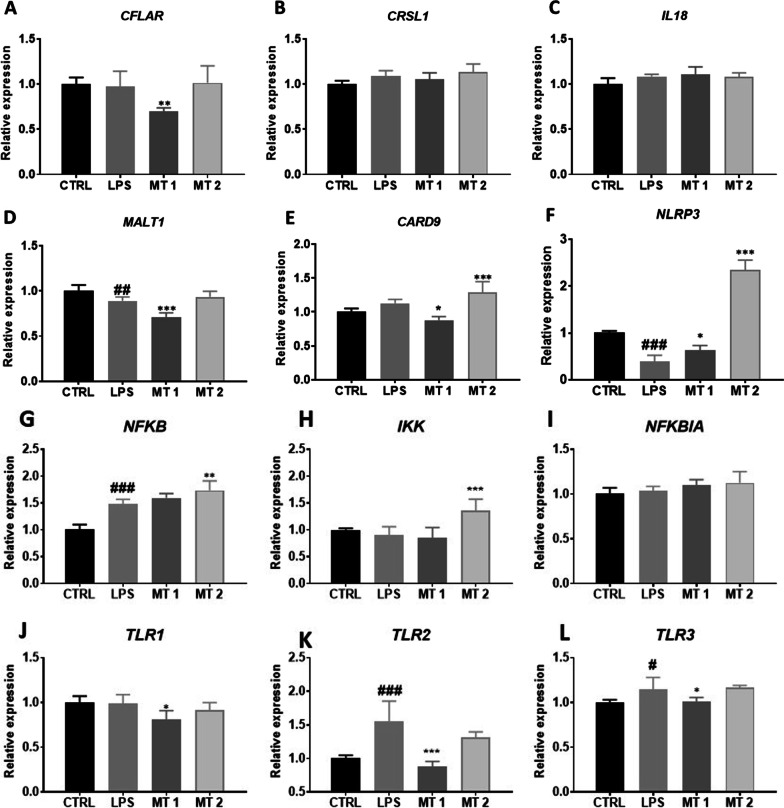
Inflammasome activation. The relative expression of CFLAR (**A**), CRSL1 (**B**), IL18 (**C**), MALT1 (**D**), CARD9 (**E**), NLRP3 (**F**), NFKB (**G**), IKK (**H**), NFKBIA (**I**), TLR1 (**J**), TLR2 (**K**), TLR3 (**L**) was investigated with RT-qPCR. Results expressed as mean Statistical significance indicated as asterisk (*) when comparing the result to LPS and as number sign (#) when comparing to CTRL. *,#*p* < 0.05, **,##*p* < 0.01, ***,###*p* < 0.001

Inflammasome activation in the cells was established with RT-qPCR. We found that cells treated with lower concentration of mitochondria (MT1) were characterised by decreased CFLAR gene expression in comparison to LPS treated group (Fig. 6A). Interestingly, higher dose of mitochondria (MT2) did not influence its expression level. No statistically significant differences were noted between groups in case of CRSL1 (Fig. 6B) and IL18 (Fig. 6C) expression. Cells from MT1 group displayed diminished MALT1 (Fig. 6D) as well as CARD9 (Fig. 6E) expression. Cells treated with mitochondria displayed elevated NRLP3 expression at both concentrations (Fig. 6F). Mitochondrial transfer at concentration MT2 significantly increased expression of NFKB (Fig. 6G) and IKK (Fig. 6H) in comparison to LPS group. No differences in the expression of NFKBIA (Fig. 6I) were found between investigated groups. TLR1 expression was significantly reduced in MT1 group in comparison to cells treated only with LPS (Fig. 6J). Mitochondria at MT1 concentration significantly downregulated the expression of TLR2 (Fig. 6K) and TLR 3 (Fig. 6L) in comparison to LPS group.

### Regulation of mitochondrial metabolism and dynamics

**Fig. 7:**
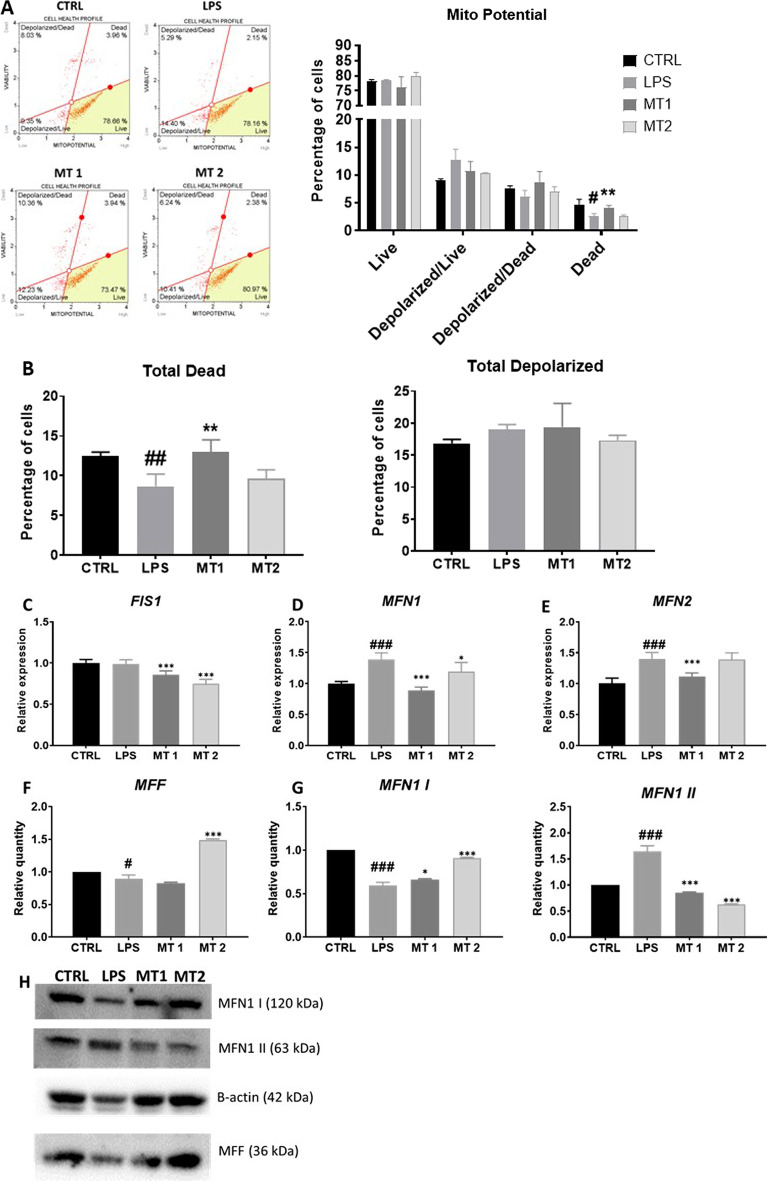

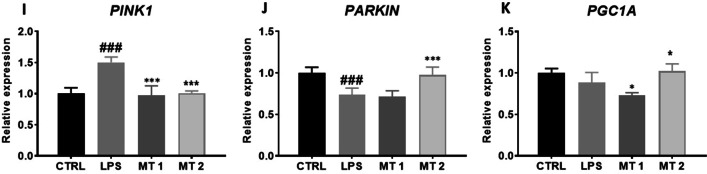
Regulation of mitochondrial metabolism and dynamics. Oxidative stress was analyzed with Muse Mitopotential kit (**A**). Based on the obtained results total dead cells (**B**) number was calculated. Relative expression of genes related to mitochondrial dynamics FIS1 (**C**), MFN1 (**D**), MFN2 (**E**) was established with RT-qPCR. The level of mitochondrial proteins MFF (**F**) and MFN1 (**G**) was investigated with western blot (**H**).  Additionally, expression of mitophagy PINK1 (**I**), PARKIN (**J**) and mitochondrial biogenesis PGC1A (**K**) genes was analysed with RT-qPCR. Results expressed as mean ± SD. Statistical significance indicated as asterisk (*) when comparing the result to LPS and as number sign (#) when comparing to CTRL. *,#*p* < 0.05, **,##*p* < 0.01, ***,###*p* < 0.001

*To evaluate oxidative stress in cells, Muse MitoPotential analysis* was *conducted* (Fig. 7A). *Interestingly, we observed* increased number *of dead cells in* MT1 *group in* comparison *to* LPS *group* (Fig. 7B)*.* Decreased *expression FIS1* (Fig. 7C), MFN1 (Fig. 7D) and MFN2 (Fig. 7E) was observed upon mitochondria treatment. To support RT-qPCR findings, the western blot analysis of protein related to mitochondrial fusion and fission were conducted Mitochondria at MT2 concentration enhanced the synthesis of MFF (Fig. 7F) in comparison to LPS group. *Obtained data showed upregulation of MFN1* I *and downregulation* of *MFN1 II* in mitochondria treated groups in comparison to LPS treatment (Fig. 7G). Representative bands are shown at Fig. 7H. Furthermore,, we have found significantly decreased expression of PINK1 in both mitochondrial treated group in comparison to LPS group (Fig. 7I) Interestingly, Parkin expression were enhanced in MT2 group (Fig. 7J) The similar phenomena were observed in PGC1A expression (Fig. 7K).

## Discussion

In recent years, mitotherapy- the idea to replace dysfunctional mitochondria with their healthy counterparts, has emerged as a novel therapeutic strategy against multiple disorders. It was shown, that restoring energetic balance in cells is able to reverse degenerative changes, improve their cytobiological properties and inhibit disease progression [16]. Current studies focus on exogenous mitochondria transplantation to recipient cells and natural horizontal mitochondrial exchange between cells via tunnelling nanotubes [11]. Both approaches result in the reprogramming of cell metabolism and plasticity. Herein, we investigated how transfer of exogenous mitochondria (MT) isolated from PBMCs into synoviocytes treated with LPS (in vitro SI experimental model) affects recipient cells metabolism. In most of the studies, mesenchymal stem cells (MSC) are chosen as a mitochondria source as it was shown in vivo, MSC transfer their mitochondria to impaired cells and stimulate regenerative process [14]. However, from the perspective of mitotherapy clinical application, MSC represents wide range of limitations which include the need of surgical procedure to harvest the tissue as well as costs and time required to isolate and culture cells. What is more, depending on patient condition and age, MSC display decreased proliferation capacity thus obtaining sufficient number of cells is impractical and expensive [17, 18]. For that reason, we decided to utilize mitochondria isolated from PBMCs as they represent more accessible source and blood can be harvest easily in relatively large amounts.

Mitochondria represents a relatively large, negatively charged organelles which limits their incorporation into recipient cells. Since now, three mechanism responsible for purified mitochondria internalisation have been proposed: micropinocytosis, CD38 pathway and heparan sulphate activation. Furthermore, it was shown, that metabolically healthy cells are somehow resistant to MT both in vitro and in vivo which highlight selectivity of this rescue mechanism. Thus mitochondria can be delivered to cultured cells by adding them to culture media and incubation, however cell cytophysiological properties are the main factor influencing the efficacy of mitochondria entry to recipient cells. In order to enhance the efficacy of intact mitochondria delivery into cultured cells several different approaches have been tested e.g. magnetic nanoparticles labelling [19], Pep-1 modification [20] and centrifugation [21].

FLS are predominant cell type in synovial intima and via interaction with each other, other cell types, extracellular matrix and other molecules regulate the homeostasis of the tissue. However, in inflamed synovium FLS as well as macrophages become activated, form a pannus-like structure, a hyperplastic synovial lining that migrate to the joint space and cartilage promoting joint destruction [3]. After activation cells switch their metabolism, change gene expression profile and in consequence acquire pathogenic phenotype. They produce large quantities of pro-inflammatory cytokines, chemokines and MMPs, display high proliferation/clonogenic potential, migrate and invade cartilage tissue. One of the prominent characteristic of activated FLS is resistance to apoptosis which most likely results from changes in mitochondrial apoptotic pathway. Previous studies have shown, that the use of extremely small dosages of intra-articular E. coli LPS can create a model of synovitis that mimics acute synovitis in horses [22]. In presented study, FLS were stimulated with LPS to establish a synovial cell inflammation model and to validate the application of mitotransfer as a strategy to reverse this damage.To our knowledge, this the first in vitro study to demonstrate that MT can be used to re-establish mitochondrial function loss in synoviocytes after LPS exposure. We hypothesised, that transfer of functional mitochondria to FLS will restore their phenotype, secretory activity and plasticity. Previous studies have shown, that MT rescues the loss of mitochondrial mass, function, and viability of cells exposed to UVR [23].

Here, we observed that, LPS-treated synoviocytes, similar to cells isolated from synovitis affected individuals, exhibited abnormal proliferation and resistance to apoptosis. Likewise, LPS exposure induced changes in mitochondrial dynamics, increased mitophagy and expression of pro-inflammatory cytokines. Under LPS treatment and synovitis.

In presented study, we have found that LPS activated synoviocytes were characterised by enhanced proliferation (Ki67/DNA content) and reduced apoptosis (BAX:Bcl2, p53, Annexin V). Results showed that synoviocytes after MT were characterised by significantly decreased Ki67 accumulation and enhanced expression of their p53 mRNA that could be associated with the induction of cell arrest or repair of mtDNA [24].

In the next step, we investigated whether improvement of mitochondrial function will reduce the synthesis and expression of pro-inflammatory mediators in recipient FLS. IL1B plays crucial role in the progression of arthritis via modulation of NF-κB-mediated ERK/STAT signalling pathway while its inhibition decrease inflammation and facilitate the treatment of disease [4]. The present results suggested that mitotransfer decrease IL1B on the mRNA and protein expression levels. What is more, previous studies have reported that the expression levels of IL6 and IL10 are downregulated in patients with rheumatoid arthritis [25] however, in our research we have found that mitotransfer significantly enhanced their amount on mRNA and protein levels. Thus we hypothesise, that inhibition of IL1B increased expression of IL6 and IL10 which indicate that therapeutic potential of mitotherapy in FLS is based on the modulation of immune response.

The MAPK and NF-κB pathways, extensively studied in FLS and critical for their activation and triggering the aggressive phenotype. These pathways also participate in the regulation of multiple metabolic events including cytokine production and cytokine action. The MAP kinases were shown to be activated in rheumatoid arthritis (RA) synovium and preclinical studies using their inhibitors were proved to be effective in animal models [26]. Pathways involved in inflammation and autoimmunity include not only those mediated by MAP but also nuclear factor-kappaB, interferon regulatory factor and Toll-like receptors, NOD-like receptors and the inflammasome, and phosphatidylinositol-3-kinases [27]. FLS act as an innate immune cells as they are able to recognise pathogens and endogenous ligands (e.g. heat-shock proteins and low-molecular-weight hyaluronan from necrotic cells) through Toll-like receptors (TLRs).

In the next step of the experiment, we investigated whether mitochondrial dynamics in recipient cells is affected by exogenous mitochondria. When exogenous mitochondria were transferred to LPS-treated FLS, the level of injury-induced, fission factor FIS1 and fusion-related MFN decreased. We hypothesized that excessive mitochondrial fission enhance inflammation and at the same time allows for removal of damaged organelles from cells. Interestingly, we have found that cells after mitotransfer displayed decreased expression of mitophagy-related gene PINK1 which may indicate that mitochondrial condition significantly improved. We also noted enhanced expression of PGC1A which is involved the regulation of mitochondrial biogenesis.

Overall, the anti-inflammatory effect of mitochondrial transfer indicated on a promising therapeutic strategy against SI as inflammation is a fundamental mechanism of its pathogenesis. However, it needs to be stated, that this study is not free of limitations. We did not obtain consistency between the dose-dependent effects of transferred mitochondria. Although we demonstrated that, exogenous mitochondria are able to modulate the recipient cells metabolism, we did not elucidate their quality before transplantation which represents a major limitation of performed study. It is crucial for further research to determine the integrity, energetical status in order to better understand their regenerative properties.

The metabolic function of adult cells decrease with age and disease. For example, during synovitis FLS switch their phenotype in order to promote disease progression. Thus, these cells represent an attractive therapeutic target to prevent its progression. Mitochondrial DNA mutations and impaired mitochondrial metabolism in FLS are well documented. Thus the idea to replace dysfunctional mitochondria with their healthy counterparts represents an attractive therapeutic strategy. In our study, we observed that LPS-treated synoviocytes after mitochondria transfer show cytophysiological changes suggesting that ex vivo manipulation of cells by artificial mitochondria transfer may provide a potential clinical solution against synovitis. Rejuvenation of cells via mitochondria transfer before their application to patient’s body may significantly benefit therapy outcome. In the next step, we would like to translate obtained results to clinical setting and address whether mitochondrial therapy could ameliorate LPS-induced synovitis in horses. To verify that hypothesis, we plan to deliver PBMC derived autologous mitochondria via intraarticular injection and investigate the therapy effectiveness of molecular and local level. Taking into consideration that, therapeutic potential of exogenous mitochondria is already supported by medical evidence [28, 29] proposed study is fully justified.

## Conclusion

This study provides the first evidence for the therapeutic effects of mitochondrial transfer in SI. We have proved, that exogenous mitochondria were successfully incorporated into LPS-treated synoviocytes. Mitochondrial transfer modulated proliferation, apoptosis and inflammation and thus may become a novel approach for the treatment of SI.

## Data Availability

The data that support the findings of this study are available from the corresponding author, upon reasonable request.

## Abbreviations

AIF: Apoptosis-inducing factor
ATP: Adenosine triphosphate
BAX: BCL-2-associated X protein
BCL-2: B-cell lymphoma 2
BSA: Bovine serum albumin
CARD9: Caspase recruitment domain-containing protein 9
Casp-3: Caspase 3
CFLAR: CASP8 and FADD-like apoptosis regulator
CFU-Fs: Colony forming unit fibroblastic assay
CRSL1: Cardiolipin synthase 1
DAMP: Damage-associated molecular patterns
DAPI: 4′,6-Diamidyno-2-fenyloindol
DMEM: Dulbecco's modified eagle's medium
FAPα: Fibroblast activation protein-α
FBS: Fetal bovine serum
FIS1: Mitochondrial fission 1 protein
FKBIA: Nuclear factor kappa-B inhibitor
FLS: Fibroblasts-like synoviocytes
GAPDH: Glyceraldehyde 3-phosphate dehydrogenase;
IKK: IkappaB kinase
IL-10: Interleukin 10
IL-15: Interleukin 15
IL-18: Interleukin 18
IL-1β: Interleukin1β
IL-6: Interleukin 6
IL-9: Interleukin 9
LPS: Lipopolysaccharide
MALT1: Mucosa-associated lymphoid tissue lymphoma translocation protein 1
MAP2K1: Dual specificity mitogen-activated protein kinase kinase 1
MAP2K2: Mitogen-activated protein kinase kinase 2
MAPK: Mitogen-activated protein kinase
MAPK14: Mitogen-activated protein kinase 14
MAPK8: Mitogen-activated protein kinase 8
MFF: Mitochondrial fission factor
MFN1: Mitofusin-1
MFN2: Mitofusin-2
MIT: Mitochondria
MLS: Macrophages-like synoviocytes
MMPs: Matrix metalloproteinases
Mn-SOD: Superoxide dismutase
MOMP: Mitochondrial outer membrane permeabilization
mROS: Mitochondrial reactive oxygen species
MSC: Mesenchymal stromal cells
MT: Mitochondrial transfer
mtDNA: Mitochondrial DNA
NFKB: Nuclear factor kappa B
NF-κB: Nuclear factor kappa-light-chain-enhancer of activated B cells
NLRP3: NLR family pyrin domain containing 3
NOD: Nucleotide oligomerization domain
OA: Osteoarthritis
p21: Cyclin-dependent kinase inhibitor 1
p53: Tumor suppressor p53
PARKIN: E3 ubiquitin ligase parkin
PBMC: Peripheral blood derived mononuclear cells
PBS: Phosphate buffer saline
PFA: Paraformaldehyde
PGC1A: Peroxisome proliferator-activated receptor-gamma coactivator
PINK1: PTEN induced kinase 1
ROS: Reactive oxygen species
SI: Synovitis
TLR1: Toll-like receptor 1
TLR2: Toll-like receptor 2
TLR3: Toll-like receptor 3
TNFα: Tumor necrosis factor
TNF-α: Tumor necrosis factor α
