# Correction: Mitochondria transfer restores fibroblasts-like synoviocytes (FLS) plasticity in LPS-induced, in vitro synovitis model

**DOI:** 10.1186/s12964-023-01187-0

**Published:** 2023-06-12

**Authors:** K. Kornicka-Garbowska, S. Groborz, B. Lynda, L. Galuppo, K. Marycz

**Affiliations:** 1 Department of Experimental Biology, Wroclaw University of Environmental and Life Sciences, Norwida 27B Street, A7 Building, 50-375 Wroclaw, Poland; 2 International Institute of Translational Medicine, Malin, Jesionowa 11, 55-114 Wisznia Mała, Poland; 3 Department of Surgical and Radiological Sciences, School of Veterinary Medicine, University of California Davis, Davis, CA USA

**Correction: Cell Commun Signal 20, 137 (2022)**

**https://doi.org/10.1186/s12964-022-00923-2**

Following publication of the original article [1], the authors reported an omission in the acknowledgements section. The acknowledgement should read as follows:

This project was supported by the Polish National Agency for Academic Exchange.

Further to this, the correspondence email address for this article has been updated to kmmarycz@ucdavis.edu.

The original article [1] has been corrected.
